# Fellowships, Grants, & Awards

**Published:** 2006-10

## Understanding Mechanisms of Health Risk Behavior Change in Children and Adolescents (R21)

The National Institute of Child Health and Human Development, the National Cancer Institute, the National Heart, Lung, and Blood Institute, the National Institute on Alcohol Abuse and Alcoholism, the National Institute on Drug Abuse, the Office of Behavioral and Social Sciences Research, and the Office of Dietary Supplements invite research grant applications that will enhance our understanding of the factors and mechanisms that determine changes in health risk behaviors during childhood and adolescence. The concept of health risk behavior change is used to encompass the evolution of specific health impairing behaviors. Of particular interest are factors and processes that influence the initiation, continuation, and/or cessation of one or more of the following health risk behaviors: 1) substance abuse, 2) inadequate exercise and poor dietary practices as they relate to being overweight or obese, and 3) intentional and unintentional injuries. The terms “health risk behaviors” and “risky behaviors” are used interchangeably.

The goal of this FOA is to promote optimal physical and mental health in children and adolescents. This may be accomplished by research to enhance our understanding of the origin, evolution, and termination of health risk behaviors and, ultimately, by the development of effective prevention and intervention strategies designed to maintain healthy behaviors and prevent health risk behaviors. Interdisciplinary research is sought to explore the biological, genetic, physiological, psychological, and social/environmental factors, and mechanisms that influence health risk behavior change in children and adolescents. A better understanding of theory-driven, causal pathways and recognition of mediators and moderators will help identify the etiology of health risk behaviors, precursors of health risk behaviors, and associated risk and resilience factors. The research findings will have the potential to inform the development of more comprehensive and effective prevention and intervention strategies in substance abuse, obesity, and injuries.

Many of the health risk behaviors of the 21st century result from voluntary behaviors such as unhealthy eating habits, the use of tobacco, alcohol, or other drugs, and the failure to be physically active, maintain a healthy weight, or use safety equipment. Health risk behaviors, once considered the result of faulty decision making, impulsive behavior, or psychosocial development, are now recognized as dynamic conditions (some with genetic predispositions) evolving across the lifespan. Much research has been done to document various types of health risk behaviors, but little research has focused on understanding the mechanisms and contextual factors responsible for behavior change. A biopsychosocial approach to the investigation of the mechanisms of health risk behavior change will explore the multiple spheres of influence provided by the individual, family, community, and society.

Childhood and adolescence are times of tremendous biological, cognitive, and psychosocial growth and development, taking children on a whirlwind trajectory from early childhood to young adulthood. Childhood and adolescence represent challenging targets for research aimed at understanding how health risk behaviors form; how they differ by individual, by ethnicity, and by gender; and the importance of genotype, phenotype, and the environment. Childhood offers a window of opportunity for the development of health-promoting behaviors, but also represents a period of vulnerability for the formation of health risk behaviors. Further, transition points between childhood and adolescence are marked by widespread developmental changes in emotional, social, and cognitive development that may contribute to the development of health-related behaviors in important ways. Health risk behaviors involve both sexes and all socioeconomic, ethnic, and racial groups.

Limited research exists on isolating process variables and causal pathways involved in the initiation, treatment, and cessation of health risk behaviors. The development of effective prevention and intervention strategies for health risk behaviors should include theory-driven models and hypotheses, and the identification and evaluation of mediators and moderators involved in the behavior change process.

The Healthy People 2010 objectives for the nation relate to improvements in the following leading health indicators: physical inactivity, overweight and obesity, tobacco and other substance abuse, and injury and violence. The goal of Healthy People 2010, with its focus on prevention, is to achieve longer and healthier lives for individuals and the general population. Accomplishing this goal will be facilitated by the development of effective prevention programs based on an understanding of the motivations and determinants of health risk behaviors throughout childhood and adolescence.

Health risk behaviors such as tobacco, alcohol, and other substance use, unhealthy dietary behaviors, and physical inactivity are evident in childhood and have far-reaching implications for individuals, families, and communities. Young people are trying illicit drugs at early ages, with almost one in three individuals having first experimented with alcohol (other than a few sips) before the age of 13 years. Alcohol consumption is a leading contributor to accidents, homicides, and suicides, the three leading causes of teen death.

One in five youths has smoked a whole cigarette and one in 10 youths has tried marijuana before 13 years of age. Cigarette smoking is the primary preventable cause of death in the United States. Most American adult deaths result from cardiovascular disease and cancer, with many of the associated risk factors being initiated during adolescence. Substance abuse and the other health risk behaviors carry an enormous price tag in the toll on life, quality of life, and economic costs.

Health risk behaviors are monitored by surveys such as the Youth Risk Behavior Survey (YRBS), which in 2001 showed that 10.5% of high school students were overweight, 67.8% did not attend physical education class daily, 28.5% had smoked cigarettes, and 78.6% had not eaten five servings of fruits and vegetables daily in the week before the survey.

During the 30 days before the survey, 14.1% of high school students had rarely or never worn a seat belt, 30.7% had ridden with a driver who had been drinking alcohol, 13.3% of students nationwide had driven a car or other vehicle one or more times after drinking alcohol, 47.1% had drunk alcohol, 17.4% had carried a weapon, and 23.9% had used marijuana. In the 2002 “Monitoring the Future” Survey, 11.3% of 12th graders were reported to have used illicit drugs other than marijuana in the month before the survey.

The United States is facing an epidemic of childhood obesity, with poor dietary behaviors and high rates of physical inactivity contributing to this preventable health condition. In 1999 in the United States, 13% of children ages 6 to 11 years and 14% of adolescents ages 12 to 19 years were overweight. The prevalence of overweight has nearly tripled for adolescents in the past two decades. And, though rising rates of overweight and obesity affect all racial, ethnic, socioeconomic, and education-level groups, they have disproportionately affected African Americans, Hispanics, and individuals from lower income and lower education brackets.

One potential complication of obesity is type 2 diabetes mellitus, a condition also increasing by epidemic proportion. Other complications likely to follow the increase in obesity and type 2 diabetes include increases in cardiovascular disease, kidney failure, and blindness. Besides type 2 diabetes, overweight and obese children and adolescents are at risk of becoming overweight adults with coronary artery disease, hypertension, stroke, respiratory problems, gallbladder disease, osteoarthritis, sleep apnea, and some forms of cancer. Obese individuals also face decreased productivity, social stigmatization, high health care costs, and premature death. Each year, 300,000 deaths in the United States are associated with obesity.

Physically inactive youth with low levels of cardiovascular fitness, high percentage of body fat, and large amounts of visceral adipose tissue have unfavorable cardiovascular risk profiles (e.g., low HDL, insulin resistance, and high triglycerides and LDL) which increase their risks of developing cardiovascular disease later in life.

Although physical activity can help prevent excessive weight gain, more than a third of all middle and high school students do not get the recommended 30 minutes of moderate physical activity on most days of the week. Regular exercise and participation in sports or physical education classes can have positive effects such as building and maintaining strong muscles and bones, controlling weight, and providing positive psychological benefits. Physical activity declines as children transition from childhood through adolescence. Children of lower socioeconomic status are less active than children of higher socioeconomic status, and girls are less active than boys during adolescence.

Unintentional injuries are the leading cause of death for individuals from age 1 to 24 years. Motor vehicle crashes, fires, and poisonings injure and kill thousands of children each year, despite the fact that a number of effective preventive strategies exist. Understanding factors that influence children’s decisions to wear seat belts and bike helmets, and identifying motivational aids to enhance their compliance with safety devices, will inform prevention and intervention strategies and subsequently reduce unintentional injuries and deaths.

For years, researchers have been intrigued by health risk behaviors and have expended extensive effort to observe, describe, predict, and prevent risky behaviors. Many health risk behaviors co-occur and co-vary in segments of the population, although the basis of the covariance is frequently unknown. Research is needed that moves beyond measuring variance in search of a comprehensive understanding of the processes and factors responsible for health risk behavior change. Numerous intervention and prevention programs have been implemented for substance abuse, physical inactivity, obesity, violence, and injury prevention, yet the effectiveness in many cases has been limited and the results have been short term. It is apparent that providing factual information concerning the dangers and negative consequences associated with health risk behaviors is not sufficient to deter all individuals from participating in high risk health behaviors. Understanding why some individuals are risk takers, the timing of their health risk behaviors, and the factors that influence their decisions and actions are all important in advancing child and adolescent health risk behavior.

Although a number of theories have been developed to explain adult behavior change, few have considered the multiple systems of influence and the complexity of their combined effects on behavior change in children and adolescents. Theoretical models relevant to health and risk behavior include self-regulation theory (Kanfer 1970), health belief model (Rosenstock 1974), theory of reasoned action (Fishbein and Ajzen 1975), subjective culture and interpersonal relations theory (Triandis 1977), protection motivation theory (Maddux and Rogers 1983), theory of planned behavior (Ajzen 1985), self-determination theory (Deci and Ryan 1985), trans-theoretical model of behavior change (Prochaska, DiClemente, and Norcross 1992), social cognitive theory (Bandura 1994), and the ecological models (Sallis and Owen 1999; Marshall and Biddle 2001).

Behavioral change theorists have identified a number of factors believed to play important roles including attitudes, intentions, skills, emotions, self-standards, self-efficacy, social norms, intrinsic and extrinsic motivation, and environment. Neuroscientists are discovering more about the complex changes in neurobehavioral systems with underpinnings that control behavior and emotion. This program announcement welcomes the behavioral, biobehavioral, and neuroscience communities to engage in the search for causal pathways to health risk behaviors.

The health risk behaviors of interest to this FOA include substance abuse, physical inactivity and poor dietary practices, and intentional and unintentional injury. These topics were selected because each represents a significant risk to the health and well-being of youth, with subsequent social and health implications during adulthood. These important health risk behaviors are amenable to short-term change in some cases and, at times, to long-term change. Additionally, these health risk behaviors have a high co-occurrence in individuals; it is important to understand factors that contribute to the development of each risky behavior independently, as well as the possible synergistic, additive, or dynamic interaction of these risk behaviors.

Studies of interest may be observational, epidemiological, interventional, secondary data analyses (i.e., analysis of existing data), and cost/benefit analyses. A multidisciplinary approach is encouraged and research findings from fields such as developmental pediatrics, psychology, behavioral science, neuroscience, neuropsychology, business, education, public policy, and others will be considered.

Health risk behaviors are influenced by a multitude of genetic, social, environmental, psychological, and physiological factors. Intrinsic factors such as genetic makeup, temperament, and memory of prior experiences combine with social and environmental factors to influence the physical, emotional, and intellectual development of an individual. Applicants are encouraged to expand on current theories of behavior change and to consider techniques, strategies, and other models such as intrinsic and extrinsic motivation, motivational interviewing, feedback interventions, contingency management, social marketing, innovation diffusion, behavioral extinction, behavioral momentum/choice theory, contingency management techniques, behavioral economics, models of decision making, and goal-directed behaviors. Numerous skills are involved in behavior change and should be considered for applications responding to this FOA. These skills include self-monitoring (self-awareness), goal setting (realistic and specific), cognitive restructuring, stress management, mental imagery, relapse prevention skills, time management, conflict resolution, assertiveness skills, decision-making skills, and substituting healthy behaviors for unhealthy behaviors. Identifying skills absent or ineffective in health risk behaviors and also those that are linked to healthy behaviors will be helpful.

Both internal and external (contextual) factors contribute to an individual’s propensity to engage in or refrain from health risk behaviors. What these factors are, how they interact, for whom, and when in the developmental trajectory, are all questions of importance in understanding health risk behaviors and behavior change.

Examples of research questions designed to fill gaps in our understanding of mechanisms and factors responsible for substance abuse, physical inactivity, and poor dietary practices, and intentional and unintentional injuries include, but are not limited to, the following:

### Biological Influences on Substance Abuse, Physical Inactivity and Poor Dietary Practices, and Injuries

1) What are the basic biological factors and processes involved in the evolution of health risk behaviors? 2) What determines for whom and when in the developmental trajectory risk behaviors occur? 3) What neurochemical factors/processes and neuroanatomical circuits are involved in health risk behavior change and how do these systems evolve over time? 4) What can newer imaging techniques contribution to our understanding of the links between neuroscience (developing brain) and behavioral science (health risk behavior)? 5) What are the bi-directional influences of physiological contributions, (neurobiological, endocrine, and immune) to risk behaviors, and how do changes to the neural, endocrine, and immune systems over the developmental trajectory influence health risk behaviors? 6) What explains the differences and similarities in risk taking behavior by age and sex? 7) What are the genetic predispositions to health risk behavior and how do resilience factors (such as parental attachment and school connectedness) attenuate genetic influences? 8) Does prenatal exposure to stress and/or teratogens (alcohol, tobacco, drugs of abuse) contribute to the offsprings propensity to participate in health risk behaviors and, if so, how? 9) How do individuals identify, evaluate, differentiate, perceive, and internalize the inherent danger of health risk behaviors? 10) How do biological and experiential influences interact in the process of health risk behavior? How do these interactions change across childhood and by sex? 11) How does communication (parent/child or peer/peer) influence health risk behaviors? 12) What interactions occur between sensation, perception of risk, emotion, learning, and memory and how do these interactions influence risk behavior? 13) What roles do physiologic states and changes induced by pain, sleep deprivation, level of arousal, and exposure to drugs or hormones play in health risk behaviors? 14) What effect does stress have on health risk behavior?

### Psychological Influences on Substance Abuse, Physical Inactivity and Poor Dietary Practices, and Injuries

1) What explains the differences and similarities in risk taking behavior by age and sex? 2) What roles do metacognitive insight, risk perception, attention, beliefs, cognition, decision making, judgment, memory, self-regulation, sensation seeking, prosocial activity, goal setting, spirituality, and moral values play in risky behavior? 3) What is the role of prior experiences in current and future health risk behavior? 4) What are the roles of aggression, impulsivity, and antisocial behavior in risk behavior? 5) Can the developmental timeline for risk factors be shortened? 6) What is the role of emotion (including reactivity and regulation) and how does the goal to elicit positive emotions or to avoid negative emotions affect an individual’s current state of mind and willingness to participate in risk behavior? 7) What is the role of orientation to present versus future in risk behavior? 8) How can researchers study risk behavior under real-world social and emotional conditions?

### Contextual Influences on Substance Abuse, Physical Inactivity and Poor Dietary Practices, and Injuries

1) What roles do contextual factors play in health risk behaviors? 2) How do parenting styles and behaviors, culture, and the socialization process influence children’s health risk behavior? 3) What bi-directional effects on risky behavior occur as a result of the clash between parenting style and child’s temperament? How are these conflicts between parenting style and child’s temperament tempered by time and experience? 4) What impact do social and contextual factors have on biological factors that influence health risk behavior? 5) Which environmental groups (peer, family, and other social groups) influence health risk behavior and how? 6) How and why do resilience factors develop and can they be promoted at earlier ages? 7) What is the role of environmental feedback in risk behavior? 8) How do environmental opportunities (gangs, cliques, money, social capital) promote or prevent risk behaviors? How critical is timing and type of opportunity? 9) What is the role of the media in promoting health risk behavior and how can this be counteracted during childhood and adolescence? 10) What are the essential elements (content, delivery, deliverer, timing) in effective health promotion messages and programs? 11) How can health promotion messages be tailored effectively for younger children? What are the roles of reinforcement (booster) messages to prevent future risk behavior?

### Co-occurring Health Behaviors

1) Which health risk behaviors co-occur and co-vary in youth and why? 2) What linkages exist between healthy and unhealthy behaviors during childhood and adolescence? 3) What are the connections between multiple unhealthy behaviors? 4) Which co-occurring mental health conditions are associated with health risk behaviors in youth and what is the relationship? 5) Are health risk behaviors more common in children with disorders of attention, emotion regulation, or learning and, if so, why?

The evolution and vitality of the biomedical sciences require a constant infusion of new ideas, techniques, and points of view. These may differ substantially from current thinking or practice and may not yet be supported by substantial preliminary data. By using the R21 mechanism, the NIH seeks to foster the introduction of novel scientific ideas, model systems, tools, agents, targets, and technologies that have the potential to substantially advance biomedical research.

The R21 mechanism is intended to encourage new exploratory and developmental research projects. For example, such projects could assess the feasibility of a novel area of investigation or a new experimental system that has the potential to enhance health-related research. Another example could include the unique and innovative use of an existing methodology to explore a new scientific area. These studies may involve considerable risk but may lead to a breakthrough in a particular area, or to the development of novel techniques, agents, methodologies, models, or applications that could have a major impact on a field of biomedical, behavioral, or clinical research.

Applications for R21 awards should describe projects distinct from those supported through the traditional R01 mechanism. For example, long-term projects or projects designed to increase knowledge in a well-established area will not be considered for R21 awards. Applications submitted under this mechanism should be exploratory and novel. These studies should break new ground or extend previous discoveries toward new directions or applications. Projects of limited cost or scope that use widely accepted approaches and methods within well established fields are better suited for the R03 small grant mechanism. Information on the R03 program can be found at http://grants.nih.gov/grants/funding/r03.htm.

This FOA will use the NIH Exploratory/Developmental Research Grant (R21) award mechanism. As an applicant, you will be solely responsible for planning, directing, and executing the proposed project.

This FOA uses just-in-time concepts. It also uses the modular budget formats (see the Modular Applications and Awards section of the NIH Grants Policy Statement. Specifically, if you are submitting an application with direct costs in each year of $250,000 or less (excluding consortium Facilities and Administrative [F&A] costs), use the PHS398 Modular Budget component provided in the SF424 (R&R) Application Package and SF424 (R&R) Application Guide (see specifically Section 5.4, Modular Budget Component, of the Application Guide).

Applicants must download the SF424 (R&R) application forms and SF424 (R&R) Application Guide for this FOA through Grants.gov/Apply.

Note: Only the forms package directly attached to a specific FOA can be used. You will not be able to use any other SF424 (R&R) forms (e.g., sample forms, forms from another FOA), although some of the Attachment files may be useable for more than one FOA.

For further assistance, contact GrantsInfo: 301-435-0714 (telecommunications for the hearing impaired: TTY 301-451-0088) or by e-mail GrantsInfo@nih.gov.

Prepare all applications using the SF424 (R&R) application forms and in accordance with the SF424 (R&R) Application Guide (MS Word or PDF).

The SF424 (R&R) Application Guide is critical to submitting a complete and accurate application to NIH. There are fields within the SF424 (R&R) application components that, although not marked as mandatory, are required by NIH (e.g., the Credential log-in field of the “Research & Related Senior/Key Person Profile” component must contain the PD/PI’s assigned eRA Commons User ID). Agency-specific instructions for such fields are clearly identified in the Application Guide. For additional information, see Tips and Tools for Navigating Electronic Submission on the front page of Electronic Submission of Grant Applications.

The application submission dates are available at http://grants.nih.gov/grants/funding/submission-schedule.htm. The complete version of this PA is available at http://grants.nih.gov/grants/guide/pa-files/PA-06-298.html

Contacts: The complete list of agency contacts is available at http://grants/nih.gov/grants/guide/pa-files/PA-06-298.

Reference PA-06-298.

## Diet Composition and Energy Balance (R01)

Overweight and obesity have increased dramatically in prevalence in the United States. More than 60% of the U.S. population is overweight (BMI > 25) and 31% meet criteria for obesity (BMI > 30). Obesity is particularly prevalent among some minority populations, such as African-American, Hispanic, and Native American women. Overweight prevalence is also increasing in children, with more than 15% of children and adolescents considered overweight and an additional 15% at risk for overweight. Obesity is associated with numerous serious and chronic diseases, including type 2 diabetes, cardiovascular disease, sleep apnea, and certain forms of cancer. The causes of obesity are complex, and involve both genetic and environmental components. Environmental changes over the past two decades have increased sedentary behaviors, decreased physical activity, and increased consumption of more energy dense foods and larger portion sizes. Although an imbalance in energy consumption and expenditure is required to promote inappropriate weight gain, the relative contributions of each to the burgeoning obesity epidemic remain in dispute.

An important gap in knowledge concerns the role of diet composition in energy balance. Diets low in carbohydrates have been purported to enhance weight loss, while those high in carbohydrates (particularly simple sugars and refined grain products) have been cited by some as contributing to weight gain. Some shorter-term studies (up to 6 months in duration) have reported greater weight loss in those who consumed a low-carbohydrate diet compared with a diet higher in carbohydrate; however, the contribution of reduced caloric consumption to weight loss with such diets has not been adequately studied. Even when carbohydrate level is similar, factors such as glycemic index, nutrient density, fiber content, and dietary variety, have also been proposed to affect energy intake, with potential relevance for enhancing attempts to promote weight loss and prevent inappropriate weight gain. In addition to carbohydrates, further research is needed to elucidate the role of other macronutrients in energy balance (including fats, proteins, and ethanol). Some fatty acids (such as conjugated linoleic acid) have been alleged to promote weight loss and/or improve body composition, although studies in humans have been inconsistent. Micronutrients such as calcium have been proposed to play a role in energy balance, either through decreased fat absorption or via other mechanisms, although research in this area is still formative. In addition, the relative impact of calcium from dairy products versus other food or supplement sources also remains in question.

The mechanisms by which diet composition affects energy balance are not well understood, but may include alternations in appetite, nutrient absorption, neuroendocrine and gastrointestinal factors, energy partitioning, physical activity, and other components of energy expenditure. Furthermore, the short- and long-term impact of diet composition on factors such as body composition, risk factors for cardiovascular disease, cancer, renal function, psychosocial function, and other health parameters is also not well characterized.

Although there have been numerous animal studies assessing the impact of diet composition on appetite and body weight, these studies have been limited by availability and use of well-defined and standardized diets. Lack of methods in laboratory practice that would permit the assessment of dietary composition and the lack of data comparing commercial preparations from various suppliers may contribute significant variability to studies designed to assess the impact of diet on energy balance. In addition to long-term studies of weight gain in response to standardized diets, short-terms studies with well-characterized diets varying in macro- and micronutrient composition would help to define the effect of diet composition on endocrine and neuronal axes involved in regulation of both food intake and energy partitioning.

This funding opportunity invites research applications investigating the role of diet composition in energy balance, including studies in both animals and humans. Collaborations between basic and clinical researchers, which explore mechanisms underlying differences in response to diet composition, are particularly encouraged. Examples of the type of research topics and approaches that would be solicited under this funding opportunity include, but are not limited to: 1) studies addressing the impact of diets varying in levels of protein, carbohydrate, fat, phytochemicals, or ethanol on appetite, food selection and intake, and energy expenditure; 2) studies examining the role of diets differing in glycemic index, dietary variety, food volume, or nutrient density on appetite, caloric intake, nutrient absorption, weight, metabolomic profiles, and body composition both short- and long-term; 3) investigation of the impact of diet composition on neuroendocrine, gastrointestinal, and other factors that may impact energy balance; 4) research assessing the impact of diets differing in macronutrient composition on psychological/behavioral function (including mood and eating-related cognitions and behaviors), bone, renal function, cardiovascular disease, and cancer risk; 5) the impact of food components such as conjugated linoleic acid (either from food or supplement sources) or calcium (either from dairy or nondairy sources, including dietary supplements) on energy balance, body composition, and disease risk; 6) studies assessing genomic and epigenomic factors underlying individual or population differences in response to diet composition that relate to chronic disease prevention; 7) brain imaging studies in humans and nonhuman primates to assess positron emission tomography, functional magnetic resonance, or cerebral blood flow imaging responses to specific dietary constituents; 8) studies to develop methods to assess dietary composition; 9) studies measuring the effects of sleep deprivation, either by experimental manipulation or due to a disease process on macronutrient consumption (e.g., Does sleep deprivation lead to changes in cravings for carbohydrate and fat? Does sleep deprivation lead to change in body weight?); 10) studies assessing dietary composition effects on the magnitude and time course of neurobehavioral and physiological responses to sleep loss, and the interaction of these effects with BMI, sex, age, and ethnicity; 11) studies assessing life-stage, racial/ethnic, and sex-related factors underlying response to diet composition, including studies in children, adolescents, and adults of various ages; 12) validation of medium or long-term effects of different dietary macronutrient composition on energy intake in community-dwelling individuals using doubly labeled water.

This funding opportunity will use the National Institutes of Health (NIH) research project grant (R01) award mechanism. As an applicant, you will be solely responsible for planning, directing, and executing the proposed project.

This funding opportunity uses just-in-time concepts. It also uses the modular as well as the nonmodular budget formats (see http://grants.nih.gov/grants/funding/modular/modular.htm). Specifically, if you are submitting an application with direct costs in each year of $250,000 or less, use the modular budget format described in the PHS 398 application instructions. Otherwise follow the instructions for nonmodular research grant applications.

The PHS 398 application instructions are available at http://grants.nih.gov/grants/funding/phs398/phs398.html in an interactive format. Applicants must use the currently approved version of the PHS 398. For further assistance contact GrantsInfo, 301-435-0714, (telecommunications for the hearing impaired: TTY 301-451-0088) or by e-mail: GrantsInfo@nih.gov.

Applications must be prepared using the most current PHS 398 research grant application instructions and forms. Applications must have a D&B Data Universal Numbering System (DUNS) number as the universal identifier when applying for federal grants or cooperative agreements. The D&B number can be obtained by calling 866-705-5711 or through the web site at http://www.dnb.com/us/. The D&B number should be entered on line 11 of the face page of the PHS 398 form.

The application submission date for this PA are available at http://grants.nih.gov/grants/funding/submissionschedule.htm. The complete version of this PA is available at http://grants/nih.gov/grants/guide/pa-files/PA-06-173.

Contacts: The complete list of agency contacts is available at http://grants/nih.gov/grants/guide/pa-files/PA-06-173.

Reference PA-06-173.

## Short-Term Educational Experiences for Research (STEER) in the Environmental Health Sciences for Undergraduates and High School Students (R25)

The National Institute of Environmental Health Sciences (NIEHS) Short-Term Educational Experiences for Research (STEER) in the Environmental Health Sciences for Undergraduates and High School Students (R25) is a component of the NIEHS Strategic Plan. The goal of the NIEHS Strategic Plan is to enable the field of environmental health sciences to have the greatest impact on preventing disease and improving human health. Through the Strategic Plan, the NIEHS is enhancing its efforts in four major areas: basic science, disease-oriented research, global environmental health, and training tomorrow’s scientists.

Motivated high school and undergraduate students are at an important juncture in their life as they consider future career choices. NIEHS seeks to provide innovative research opportunities for students in environmental health. This educational research-oriented experience is designed to attract talented high school students interested in science and undergraduates with scientific majors relevant to the environmental health sciences to graduate research careers in the environmental health sciences. The R25 educational programs submitted in response to this announcement are expected to propose an organized short-term program of research experiences and informational exchange designed to impart to participants an appreciation of research on the environmental impacts on human health. It is required that the program will focus on those environmental factors which are within the mission area of the NIEHS (see below).

An increasing concern of the NIEHS is the so-called “pipe-line” issue—that is, attracting talented high school students and science undergraduates to graduate research careers in the environmental health sciences. This issue is particularly critical for attracting students to research in the environmental health sciences since the relevant topics are rarely covered in the disciplinary undergraduate curricula. Many undergraduate curricula offer courses and programs in environmental studies, but these cover mainly ecology and the earth sciences, and offer little detail of human health impact following environmental exposure and mechanisms by which this occurs. It is hoped that by offering such an introduction to high school students and science undergraduates through short-term summer programs, the NIEHS can both increase the number and elevate the credentials of the pool of applicants to graduate programs in the environmental health sciences.

Educational programs supported by this Funding Opportunity Announcement are expected to consist of both an introduction to environmental health sciences research through experience on a research project with the participating faculty and a summer program of organized educational experiences designed to acquaint the participants with the larger field of the environmental health sciences. The focus of both the laboratory experience and the educational experiences/seminars should be on human health aspects of environmental exposure. Programs that propose only the laboratory experience within the context of a university wide summer program for undergraduates and/or high school students will not be considered responsive to this announcement.

Sudent research projects supported by these education grants should have a defined focus in the environmental health sciences, and be responsive to the mission of the NIEHS, which is distinguished from that of other institutes by its support of research programs seeking to understand how environmental exposures alter biologic processes and affect the risk of either disease development or the distribution of disease in populations. Examples of environmental exposures relevant to the mission of the NIEHS include industrial chemicals or manufacturing byproducts, metals, pesticides, herbicides, air pollutants, and other inhaled toxicants, particulates or fibers, fungal or bacterially derived toxins due to ambient exposures. Agents considered to belong to the mission area of other NIH Institutes include alcohol, chemotherapeutic agents, ionizing radiation, drugs of abuse, pharmaceuticals, smoking (except secondhand smoke), and infectious or parasitic agents, except when these are disease co-factors with an environmental toxicant exposure to produce the biological effect. Training in ecology, ecologic or microbial biotransformation, ecologic biodegradation and remediation, ecological monitoring, wildlife and fisheries biology or studies of sentinel species, geochemistry, and other ecologically based environmental studies should not be a component of this educational program. Projects in veterinary medicine where the end point is animal health or in food science are also not be included in this educational program. Projects in exposure assessment should concentrate on exposure biology, which is at the interface of exposures and human health, and research centered on biomarkers as indicators of body burden, pathophysiological changes, or inception/progression of disease, rather than environmental measurement of ambient contact or point of exposure.

The proposed research education program may complement other, ongoing research training and education occurring at the applicant institution, but the proposed educational experiences must be distinct from those research training and research education programs currently receiving federal support. The R25 is not a substitute for an institutional research training program (T32) and can not be used to circumvent or supplement Ruth L. Kirschstein National Research Service Award (NRSA) mechanisms.

This FOA will use the NIH Research Education Grant (R25) award mechanism. As an applicant, you will be solely responsible for planning, directing, and executing the proposed project.

This FOA uses just-in-time concepts. It also uses the nonmodular budget format. Applicants must complete and submit budget requests using the SF424 Research and Related (R&R) Budget Component found in the application package for this FOA.

At this time, it is not known if competing renewal (formerly “competing continuation”) applications will be accepted and/or if this FOA will be reissued.

Applicants must download the SF424 (R&R) application forms and SF424 (R&R) Application Guide for this FOA through Grants.gov/Apply.

Note: Only the forms package directly attached to a specific FOA can be used. You will not be able to use any other SF424 (R&R) forms (e.g., sample forms, forms from another FOA), although some of the “Attachment” files may be useable for more than one FOA.

For further assistance, contact GrantsInfo: 301-435-0714 (telecommunications for the hearing impaired: TTY 301-451-0088) or by e-mail: GrantsInfo@nih.gov.

The letter of intent receipt date for this RFA is 11 December 2006, with the application of receipt date 11 January 2007. The complete version of this RFA is available at http://grants.nih.gov/grants/guide/rfa-files//RFA-ES-06-009.html.

Contact: Michael C. Humble, Division of Extramural Research and Training, National Institute of Environmental Health Sciences, PO Box 12233, EC-23, 111 T. W. Alexander Drive, Research Triangle Park, NC 27709 USA, 919-316-4621, Fax: 919-541-5064, e-mail: humble@niehs.nih.gov.

Reference RFA-ES-06-009.

## U.S. Student Fellowships for Environmental Fields of Study

The U.S. Environmental Protection Agency (EPA), as part of its Science to Achieve Results (STAR) program (see http://es.epa.gov/ncer/rfa/2007/2007_star_fellow.html) and the Greater Research Opportunities (GRO) program (see http://es.epa.gov/ncer/rfa/2007/2007_star_gro_grad.html), is offering graduate fellowships for master’s- and doctoral-level students in environmental fields of study. The deadline for receipt of pre-applications is 28 November 2006. Subject to availability of funding, the Agency plans to award approximately 65 new fellowships by 20 July 2007. Master’s-level students may receive support for a maximum of 2 years. Doctoral students may be supported for a maximum of 3 years, usable over a period of 4 years. The fellowship program provides up to $37,000 per year of support per fellowship.

The U.S.EPA, as part of its GRO program (see http://es.epa.gov/ncer/rfa/2007/2007_star_gro_undergrad.html), is offering undergraduate fellowships for bachelor level students in environmental fields of study. The deadline for receipt of pre-applications is 29 November 2006. Subject to availability of funding, the agency plans to award approximately 15 new fellowships by 20 July 2007. Eligible students will receive support for their junior and senior years of undergraduate study and for an internship at an EPA facility during the summer between their junior and senior years. The fellowship provides up to $17,000 per year of academic support and up to $7,500 of internship support for a 3-month summer period.

